# Cytology-based Screening for Anal Intraepithelial Neoplasia in Immunocompetent Brazilian Women with a History of High-Grade Cervical Intraepithelial Neoplasia or Cancer

**DOI:** 10.1055/s-0042-1743163

**Published:** 2022-08-08

**Authors:** Vivian de Oliveira Rodrigues Brum, Alessandra de Souza Oliveira Tricoti, Gabriel Duque Pannain, Denise Gasparetii Drumond, Isabel Cristina Gonçalves Leite

**Affiliations:** 1 Lower Genital Tract Pathology and Colposcopy Service, Gynecology Department, Hospital Universitário da Universidade Federal de Juiz de Fora (UH-UFJF), Juiz de Fora, MG, Brazil

**Keywords:** anal cancer, high-grade squamous intraepithelial lesion, human papillomavirus, screening, câncer anal, lesão intraepitelial escamosa de alto grau, vírus do papiloma humano, triagem

## Abstract

**Objective** To determine the prevalence and possible variables associated with anal intraepithelial neoplasia and anal cancer in immunocompetent women with high-grade cervical intraepithelial neoplasia.

**Methods** A cross-sectional study involving immunocompetent women with a histological diagnosis of high-grade cervical intraepithelial neoplasia and cervical cancer, conducted between January 2016 and September 2020. All women underwent anal cytology and answered a questionnaire on characterization and potential risk factors. Women with altered cytology were submitted to anoscopy and biopsy.

**Results** A total of 69 women were included in the study. Of these, 7 (10.1%) had abnormal anal cytology results: (high-grade lesion, atypical squamous cells of undetermined significance, and atypical squamous cells, cannot exclude high-grade lesions: 28,5% each; low grade lesion: 14,3%). Of the anoscopies, 3 (42.8%) showed alterations. Of the 2 (28,5% of all abnormal cytology results) biopsies performed, only 1 showed low-grade anal intraepithelial neoplasia. The average number of pregnancies, vaginal deliveries, and abortions was associated with abnormal anal cytology. However, the highest mean regarding the cesarean sections was associated with normal cytology.

**Conclusion** The prevalence of anal intraepithelial neoplasia was compatible with data from recent studies, especially those conducted in Brazil. Opportunistic screening for anal intraepithelial neoplasia in this high-risk population should be considered. Anal cytology is suitable for this purpose, due to its low cost and feasibility in public health services.

## Resumo

**Objetivo** Determinar a prevalência e as possíveis variáveis associadas à neoplasia intraepitelial anal e ao câncer anal em mulheres imunocompetentes com neoplasia intraepitelial cervical de alto grau.

**Métodos** Estudo transversal em mulheres imunocompetentes com diagnóstico histológico de neoplasia intraepitelial cervical de alto grau e câncer cervical, feito entre janeiro de 2016 e setembro de 2020. Todas as mulheres foram submetidas a citologia anal e responderam a um questionário de caracterização e potenciais fatores de risco. Mulheres com citologia alterada foram submetidas a anuscopia e biópsia.

**Resultados** No total, 69 mulheres foram incluídas no estudo. Destas, 7 (10,1%) tiveram resultados anormais de citologia anal (lesão de alto grau, células escamosas atípicas de significado indeterminado, e células escamosas atípicas, não se pode excluir lesões de alto grau: 28,5% cada; lesão de baixo grau: 14,3%). Das anuscopias, 3 (42,8%) demonstraram alterações. Das 2 biópsias realizadas, apenas 1 apresentou neoplasia intraepitelial anal de baixo grau. O número médio de gestações, partos vaginais e abortos estava associado à citologia anal anormal. No entanto, a maior média de partos cesáreos estava associada à citologia normal.

**Conclusão** A prevalência de neoplasia intraepitelial anal foi compatível com dados de estudos recentes, principalmente daqueles feitos no Brasil. O rastreamento oportunista para neoplasia intraepitelial anal nesta população de alto risco deve ser considerado. A citologia anal é adequada para esse fim, devido ao seu baixo custo e viabilidade nos serviços públicos de saúde.

## Introduction

Anal cancer is a rare occurrence in the general population, but its incidence and mortality have been progressing worldwide since 1975, reaching 1 to 2 per 100 thousand individuals. In women, the prevalence is even higher: 2.06 per 100 thousand individuals. 1 2 In 2018, the World Health Organization 3 (WHO) estimated 48,541 new cases worldwide. In the United States, the incidence in the same year was of 1.8 per 100 thousand inhabitants, totaling 8,580 new cases, which represented 0.5% of all cancer cases in the country. 4 In Brazil, according to the latest estimates from the National Cancer Institute (Instituto Nacional de Câncer, INCA, in Portuguese), there were 408 deaths from the disease in 2015; of these, 258 were women. 5

There is strong evidence indicating that persistent infection with an oncogenic type of human papillomavirus (HPV) is a necessary condition for the development of cervical cancer, and it is one of the factors associated with the increased incidence of squamous cell carcinoma in other regions of the lower genital tract, particularly the anal canal. 6 Infection by HPV is estimated to be present in 80% to 90% of cases of anal cancer. 7 8

Other risk factors for anal cancer include: infection by the human immunodeficiency virus (HIV), being a homosexual man, engaging in receptive anal sex, the number of lifelong sexual partners, smoking, and a history of squamous cervical intraepithelial neoplasia. 1 A recent meta-analysis 9 found a 14-fold increased risk of anal cancer in patients with a previous history of vulvovaginal or cervical cancer, revealing an underestimated risk group on whom few studies have been published, 10 11 12 13 14 with prevalence rates ranging from 9% to 19%.

Guidelines for the screening of premalignant anal lesions have not yet been standardized. 1 However, as anal cancer has etiopathogenic similarities with cervical cancer, and considering the success of cervical cancer screening programs, anal cancer screening is recommended by some American and Brazilian societies, including the American Cancer Society and the Brazilian Federation of Gynecology and Obstetrics Associations (Federação Brasileira das Associações de Ginecologia e Obstetrícia, FEBRASGO, in Portuguese). 7 15 16

Currently, anal cytology is the main screening method for anal cancer. 1 It is estimated to have sensitivity and specificity similar to those of cervical cytology when performed by experienced professionals, in addition to being effective and low-cost. 13 17 High-resolution anoscopy (HRA) is the gold standard to diagnose high-grade anal lesions after an abnormal cytology result. 18 It offers greater diagnostic accuracy, by enabling the perfomance of biopsies for histological evaluation; however, it is a time-consuming examination that requires a greater learning curve, which limits its use as a primary screening method. 19

To date, most studies have focused on men who have sex with other men and people with HIV, 10 but not on immunocompetent women with high-grade cervical lesions caused by HPV. Thus, the aim of the present study is to determine the prevalence of anal intraepithelial neoplasia and anal cancer in immunocompetent women with a history of high-grade cervical intraepithelial neoplasia or cancer, using anal cytology as the primary screening method, as well as to identify the associated factors.

## Methods

Patients of the Lower Genital Tract Pathology and Colposcopy Service of the Gynecology Department at Hospital Universitário da Universidade Federal de Juiz de Fora (HU-UFJF) with a histological diagnosis of high-grade cervical intraepithelial neoplasia (glandular or squamous) or cervical cancer between January 2016 and September 2020 were invited to participate in this cross-sectional study.

The exclusion criteria were as follows: HIV infection, current pregnancy, previous or current pelvic radiotherapy, previous or current colorectal cancer, presence of anal tumor on digital rectal examination, and use of immunosuppressive drugs.

All women underwent anal cytology. They also answered a questionnaire, through a brief interview, about their main sociodemographic characteristics, sexual and reproductive history, and life habits.

Anal cytology was performed using an endocervical brush introduced up to 4 cm into the anal canal, with 360° rotation. The material was placed on a glass slide and a liquid alcohol-based fixative was applied to it. The HU-UFJF Pathology Service analyzed all samples using Papanicolaou staining. All cytological specimens were processed on glass slides (conventional cytology) and analyzed by the same pathologist.

The results followed the nomenclature recommended by the Bethesda System for Reporting Cervical Cytology: low-grade squamous intraepithelial lesions (LSIL), high-grade squamous intraepithelial lesions (HSIL), atypical squamous cells of undetermined significance (ASC-US), atypical squamous cells –cannot exclude HSIL (ASC-H), squamous cell carcinoma (SCC) or adenocarcinoma in situ (AIS). Unsatisfactory results were repeated according to the patients' agreement.

Patients with abnormal anal cytology were subsequently submitted to HRA. Before the HRA, a digital rectal examination (DRE) was performed to identify possible tumors, stenosis or thickening in the anal canal, which would contraindicate the HRA. All examinations were performed by the same experienced professional. In the HRA, the anal canal is visualized with image magnification through a colposcope, a device similar to a binocular microscope, which has a magnifying lens and a light focus, in an attempt to identify anal lesions. A rigid speculum, the anoscope, is introduced in the anus, and a diluted acetic acid solution (at 5%) is applied to observe the transformation zone between the columnar epithelium of the inferior rectum (of endodermal origin) and the squamous epithelium of the anal canal (of ectodermal origin) and look for lesions, in a manner homologous to gynecological colposcopy. Then, to refine the examination, an iodine-iodide solution (Schiller solution) was also applied. Finally, all suspicious areas were submitted to biopsy at the same time as anoscopy and sent to the laboratory for analysis.

Patients with a histological diagnosis of anal lesions were referred for multidisciplinary follow-up at the Proctology Service of the hospital.

The present study was approved by the Ethics in Research Committee of HU-UFJF.

Descriptive statistics (absolute and relative frequencies) and central tendency (mean, standard deviation [SD], and median and interquartile range) were presented. The measures of prevalence of anal lesions and their association with sociodemographic, clinical, reproductive and lifestyle variables were studied using the Chi-squared test. The agreement between the diagnostic methods of anal cytology and anoscopy was performed through the Kappa analysis.

The statistical analysis was performed using the Statistical Package for the Social Sciences (IBM SPSS Statistics for Windows, IBM Corp., Amronk, NY, United States) software, version 21.0, and values of *p* < 0.05 were considered statistically significant.

## Results

### Patient Characteristics

**Fig. 1:**
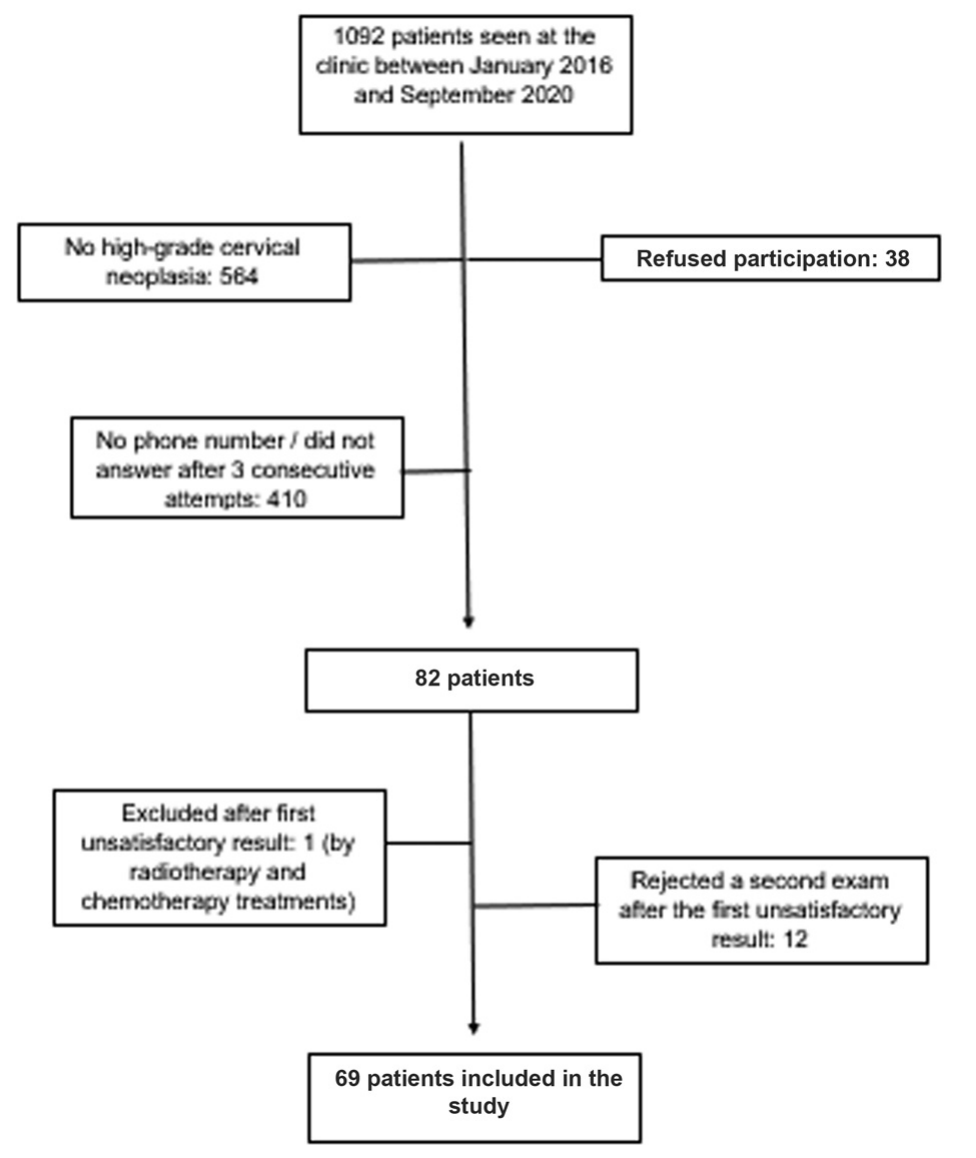
Flow chart indicating the process of patient selection.

Of the 82 women who met the inclusion criteria and agreed to participate, 69 were included in the study, as shown in Fig. 1 . We excluded 1 (1.2%) patient, as she had started radiotherapy and chemotherapy during the data collection period, and 12 (14.6%) were excluded since their first cytology result was unsatisfactory and they refused to undergo a second examination. The mean(±SD) age of the participants was 37.92(±10.15) years, most of them were white (40.6%), and in a current stable relationship (79.7%). The mean(±SD) age at the first sexual intercourse was of 16.62(±2.34) years, and the average of partners throughout life was of 8.97(±11.16). The most frequent previous cervical lesion was high-grade intraepithelial neoplasia (cervical intraepithelial neoplasia grade III, CIN III – 71%). Only 4 (5.8%) patients had cervical cancer, 3 (4.3%) with invasive squamous carcinoma of the cervix, and 1 (1.4%) with invasive adenocarcinoma. The main method of diagnosis of the cervical lesions was incisional biopsy (72.46%). The cervical lesion was a random finding in one hysterectomy for fibroids and one polypectomy. The most common treatment for cervical lesions was loop electrosurgical excision procedure (LEEP; 72.47%), followed by classic surgical conization (7.2%). The mean(±SD) interval between the diagnosis of cervical lesion and the conclusion of the study was of 19.56(±15.84) months, and there was no difference between the groups regarding normal and abnormal cytology results. Other patient characteristics are shown in Table 1 .

**Table 1:** Patient Characteristics, according to cytology results (n = 69)

Characteristics	Normal anal cytology	Abnormal anal cytology	
	n (%)	n (%)	
**Smoking history**			
Current smoker	10 (16,1)	1 (14,3)	
Never smoked	49 (79)	4 (57,1)	0,071
Previous smoker	3 (4,8)	2 (28,6)	
**Current illicit drug use**			
Yes	2 (3,2)	0 (0)	0,806
**Previous sexually transmitted disease**			
Yes	6 (9,7)	0(0)	0,513
**Anal intercourse**			
Yes	46 (74,2)	6 (85,7)	0,445
**Anal itching (current or previous)**			
Yes	18 (29)	1 (14,3)	0,372
**Anal condyloma (current or previous)**			
Yes	9 (14,5)	0 (0)	0,358
**Regular condom use**			
Yes	16 (25,8)	2 (28,6)	0,592
**Syphillis (current)**			
Yes	2 (3,6)	0 (0)	0,789
No	59 (96,4)	7 (100)	

### Prevalence of Anal Intraepithelial Neoplasms and Association with Clinical Characteristics

**Fig. 2:**
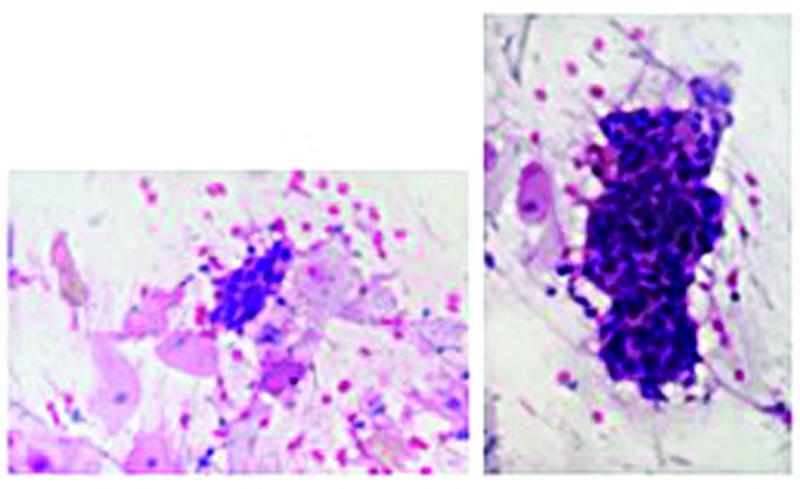
HSIL anal cytoloty (200x and 400x). High-grade squamous intraepithelial lesion showing dense squamous epithelial cellularity with pleomorphism and nuclear hyperchromasia, in addition to occasional atypical mitoses. Pap smear.

Among the 69 patients, 7 (10.1%) showed changes in the result of the anal cytology. The most prevalent changes were ASC-US, ASC-H and HSIL (28,5% each). Of the performed anuscopias, 3 (42.8%) showed changes, representing 4.3% of the total of patients. Only two biopsies were performed, since one of the patients was pregnant at the time of the examination, and we decided to postpone the biopsy until after delivery. One biopsy showed normal results, and the other showed low-grade intraepithelial neoplasia. Anal cancer was not found. There was no statistically significant difference between the normal and altered anal cytology groups in relation to sociodemographic variables, lifestyle, presence of itching or anal bleeding, sexual behavior, and age at the first sexual intercourse. There were differences between both groups in relation to all gestational variables. The mean number of pregnancies was higher in the altered anal cytology group (2.57 ± 2.63) than in the normal anal cytology group (1.66 ± 1.44) ( *p* = 0.01). The mean number of vaginal deliveries was also higher in the altered anal cytology group (2.14 ± 1.95) compared with the group without alteration (0.98 ± 1.24) ( *p* = 0.05), as well as the mean number of spontaneous abortions (0.41 ± 0.78 versus 0.18 ± 0.49 respectively; *p* = 0.05). In contrast, the highest mean number of cesarean sections was associated with the normal anal cytology group (0.5 ± 0.7 versus 0.0 in the altered group; *p* < 0.01). The association of cytology and anoscopy results is shown in Fig. 2 .

## Discussion

In the present cross-sectional study, 10.1% of immunocompetent women with a history of high-grade cervical intraepithelial neoplasia or cervical cancer had abnormal cytology.

Many studies have assessed the prevalence of anal intraepithelial neoplasia in subgroups that are already known to be at a higher risk, such as men who have sex with other men, or those with HIV. 10 However, the present is one of the few Brazilian studies that evaluated the prevalence of anal intraepithelial neoplasia based on anal cytology in immunocompetent women with a history of previous cervical lesion caused by HPV.

In the present study, the prevalence of abnormal anal cytology was similar to that of another Brazilian study 20 that evaluated the same population: 11.4%. However, unlike the present study, it also included patients with low-grade cervical intraepithelial neoplasia. Previous studies 10 11 12 13 14 show similar rates, ranging from 9% to 19%. In contrast, a recent Canadian study found a prevalence of 30.3% of cytological abnormalities among the same population. One reason for this discrepancy may have been that the percentage of women with a history of cervical cancer was higher than that of our study (33.8% versus 5.8% respectively). However, there was no statistically significant difference in the prevalence of anal intraepithelial neoplasia among women with cervical cancer or cervical intraepithelial neoplasia. 17

The present study obtained 15.85% of unsatisfactory cytological results in the first anal cytology specimen collection, and the literature 21 points out that a rate between 9% to 17% is acceptable. Anal cytology is performed blindly, without the use of an anoscope, 22 which may be one of the reasons behind the rates of unsatisfactory results. This is significant, since 13 patients were excluded from the original sample of 82 women, 12 of them due to refusal to undergo a second sample collection.

We found an association among the mean number of pregnancies, vaginal deliveries and abortions, and abnormal anal cytology. In Brazil, women with a higher number of pregnancies usually have low socieconomic status and low level of schooling, factors sometimes associated with a higher risk of acquiring HPV infection. 23 A Brazilian study 24 similar to the present study also found an association between parity and the prevalence of anal HPV. However, this is an unusual finding in the literature. One study 25 evaluated the prevalence of anal intraepithelial neoplasms in women with a history of cervical HPV, but found no association between abnormal anal cytology, and the numbers of pregnancies and abortions. Neither was parity associated with anal cytological abnormalities in another more recent Brazilian study. 20 These two studies did not classify pregnancies regarding the number of vaginal or cesarean deliveries. 20 25 Other similar studies 1 17 26 failed to assess the parity variable.

A 2013 study 27 which only evaluated issues related to parity and HPV found an association between vaginal births and intraepithelial cervical neoplasia. Possible mechanisms would be the local tissue damage that occurs during vaginal delivery or cellular oxidative stress, which are more likely to damage DNA and facilitate HPV integration. 28 29 As HPV infection can also spread from the genital to the anal region, 30 these mechanisms may justify the association between vaginal delivery and abnormal anal cytology found in the present study. However, these mechanisms are still controversial, since it is also possible that the inflammatory reaction secondary to local trauma by vaginal delivery may induce regression of the lesions caused by HPV. 31

In contrast, the number of cesarean sections was associated with normal anal cytology. The performance of caesarean delivery is lower in the Brazilian Unified Health System, than in the private health sector, showing a direct correlation with higher socioeconomic status and, consequently, less risk of HPV infection. 32

The practice of anal sex reported by the patients was significant (75.4%), a prevalence considerably higher than that reported among American women between 15 and 44 years of age (33.2%). 33 However, it was not associated with cytological abnormalities in the present study. This finding has been corroborated by similar studies. 17 20 34 Although the practice of anal sex is a risk factor for anal intraepithelial neoplasia, 35 36 the finding of the present study corroborates the idea that HPV infection can also spread from an area to another, being able to originate in genital organs and extend to other areas, such as the anal canal. 30

No association was found regarding abnormal anal cytology and other variables, such as smoking, drinking, use of illicit drugs, use of condoms, number of sexual partners, and presence of pruritus or anal bleeding, which is in line with similar studies. 1 25 37 One of the reasons is the fact that the sample of the present study was homogeneous regarding these characteristics.

In general, the result of the HRA was normal in 57.1% of the cases of altered cytology. The decrease in agreement between the examinations can be explained by certain factors. In the anal canal, there is an extensive squamous-columnar junction associated with deep crypts that enable cytology to identify lesions that remain imperceptible to HRA, leading to false-negative results. 38 On the other hand, keratinization, a common process of the anal epithelium, can cause cell desquamation, with the possibility of underestimation of the lesion or the obtainment of false negatives in cytology samples. 39

The only case of biopsy-proven anal intraepithelial neoplasia, a low-grade neoplasia, came from a low grade anal cytology, which corroborates the hypothesis that anal cytology is less discriminating regarding high-grade lesions. 22 The only two cases of biopsy-proven anal lesions in a 2017 study 1 also came from cytologies reports of minor findings, such as ASC-US.

Although there is no data yet that demonstrates that the identification and treatment of high-grade anal squamous intraepithelial neoplasia leads to a reduced risk of developing anal cancer, a recent review 40 by a group of experts from the American Society for Colposcopy and Cervical Pathology and the International Anal Neoplasia Society suggested that women with neoplasia of the lower genital tract may be considered for screening through anal cytology. In Brazil, this recommendation was made by FEBRASGO in guidelines published in 2010. 16 In our study, 84.14% of the participants completed the protocol and were curious to understand more about the subject. As they already did or, had already undergone a rigorous follow-up, of at least 2 years, of the cervical lesions, this opportunistic screening of anal lesions based on anal cytology proved to be efficient.

One of the limitations of the present study was that only patients with abnormal anal cytology were submitted to HRA. The literature 41 points out that the concomitant performance of the two examinations can increase the probability of detecting anal intraepithelial lesions. Another limitation was the lack of patient follow-up. In those with abnormal cytology and first normal HRA, the ideal would be to perform at least a second HRA for diagnostic confirmation. In addition, the characteristics of the study do not enable conclusions to be drawn about which lesion developed first (that of the cervix or that of the anal canal) or for how long these lesions coexisted.

## Conclusion

The prevalence of 10,1% of intraepithelial neoplasia found in the present study was compatible with the prevalence found in recent studies. Parity and the numbers of vaginal deliveries and abortions were associated with abnormal anal cytology. The number of cesarean sections was associated with normal cytology. Despite being uncommon findings, they are associated, in Brazil, with income inequality and poor access to health services, reflecting a pattern already known for cervical intraepithelial lesions and cervical cancer. This reinforces the need for greater medical care for this population, that is most vulnerable to the effects of HPV. Opportunistic screening for anal intraepithelial neoplasia in patients with high-grade cervical intraepithelial neoplasia should be considered, especially in immunocompetent women. The study showed that anal cytology proved to be an adequate test for screening, mainly due to its low cost and availability in the public health service. Although the agreement between the diagnostic methods has been shown to be weak, anal cytology at least enables the identification of HPV-induced lesions, facilitating referral to anoscopy and biopsy. It also promotes public awareness and education regarding a problem that was previously ignored.
